# Can hepatology lead the way? Synthesising current global curricula and proposing a model for an international specialty curriculum

**DOI:** 10.1016/j.clinme.2026.100615

**Published:** 2026-07-06

**Authors:** James C. Liu Yin, Matthew Parsons, Morro M.L. Touray, James O’Beirne, Ponsiano Ocama, Osman Dar, Ajay Duseja, Sanjaya Satapathy, Thomas Schiano, Aftab Ala

**Affiliations:** a School of Immunology and Microbial Sciences, King’s College London, London, UK; b The Roger Williams Institute of Liver Studies, King’s College London, London, UK; c King’s College Hospital NHS Foundation Trust, Denmark Hill, London, UK; d Faculty of Health and Medical Sciences, University of Surrey, Guildford, UK; e School of Medicine, Cardiff University, Heath Park, Cardiff, UK; f Department of Gastroenterology and Hepatology, Sunshine Coast University Hospital, Birtinya, QLD, Australia; g Gastroenterology Unit, Department of Internal Medicine, Makerere University College of Health Sciences, Kampala, Uganda; h Office of the Director General, Africa Centres for Disease Control and Prevention, P.O. Box 3243, Addis Ababa, Ethiopia; i Department of Hepatology, Postgraduate Institute of Medical Education and Research, Chandigarh, India; j Division of Hepatology, Sandra Atlas Bass Center for Liver Diseases and Transplantation, Donald and Barbara Zucker School of Medicine, Northwell Health, Manhasset, NY, USA; k Recanati/Miller Transplantation Institute, Icahn School of Medicine at Mount Sinai, New York, NY, USA

**Keywords:** Education, Training, Curricula development, Training assessment, Framework, Improving care, Initiatives

## Abstract

**Background:**

Increased understanding and treatments for liver disease have led in recent decades to the emergence of hepatology as a subspecialty within gastroenterology, and as a standalone medical specialty. Growing challenges in hepatology are a global problem, yet no global framework for education and training has been established. We therefore aim to conduct feasibility work to compare existing international curricula, examine the ability to merge existing frameworks and hypothesise a basis for a shared curriculum leading to a standardised global accreditation.

**Method:**

We employed a cross-sectional content mapping approach to assess the hepatology training of eight regions across five continents. Regions were selected that had an English language curriculum to allow accurate comparison. Data were gathered for entry requirements, training pathway, research and procedural elements. These were structured using an established framework allowing for direct comparison to be drawn.

**Results:**

Endoscopic competency for oesophago-gastroduodenoscopy (OGD) is the only procedural requirement shared between all regions that is necessary for completion of hepatology training. Trainees are assessed through a combination of clinical-based discussions / clinical examinations (CBD/CEX), direct observation of practice (DOP), logbooks, supervisor evaluations and oral examination using at least two of these methods.

**Conclusion:**

We found areas of considerable homogeneity between the eight curricula examined, which would be favourable and practical to develop a shared curriculum. Where differences exist, they may reflect the variation in local medical requirements. By adopting a competency-based assessment system, we believe there is the capacity to develop a global curriculum without compromising the quality of hepatology training. We propose a possible framework and areas of focus to establish the minimum standards to provide global uniformity in hepatology training and assessment while allowing for additional competencies to adapt the curriculum to local exigencies.

## Highlights

• Liver disease remains a major global cause of illness and death.
• Hepatology is a recognised specialty worldwide with varied training routes.
• We mapped hepatology curricula across eight regions on five continents.
• Shared themes suggest feasibility of a unified global competency framework.
• A global curriculum may ease accreditation barriers and specialist mobility.

## Introduction

Hepatology has evolved rapidly as a distinct specialty, having historically been embedded within gastroenterology training. Over time, with an improving understanding of different liver diseases, the development of liver transplantation, rising global burden of liver-related mortality and morbidity, the need for dedicated specialist training in this field has become apparent.^1^

Seismic shifts in hepatology now mean that liver disease is one of the few diseases with a rising mortality trend in high-income countries (HIC).^1^, ^2^ Rising childhood and adult obesity, sedentary lifestyle, ultra-processed foods, excessive alcohol consumption in both HIC and low-income countries (LIC) are significant risk factors for liver disease and are posing significant challenges for hepatologists,^3^ paralleled by the high prevalence of viral hepatitis and rising hepatocellular carcinoma (HCC) rates; these continue to be healthcare and public health challenges.^4^

Consequently, paradigm shifts in liver disease management away from reactive secondary and tertiary care towards proactive prevention strategies delivered in community and primary care settings have been encouraged,^1^ particularly relevant as hepatology shifts more towards integrated community-based care models.

While there are already global initiatives within hepatology, such as the World Health Organization (WHO)’s aim for elimination of viral hepatitis C by 2030,^5^ there are no such initiatives to standardise and unify hepatology training and education across the WHO member states.^6^ The COVID-19 pandemic highlighted the global response to complex health challenges, where industry, governments, healthcare systems and clinicians were necessary to modulate this multifaceted disease. In today’s rapidly evolving medical landscape, continuous adaptation is crucial to enhance health outcomes and reduce health inequality both locally and globally. Research highlights the influence of these lessons on shaping national health policies^7^ and reinforcing the importance of standardised adaptable training models. Our consortium believes that training is the appropriate mechanism to effect significant change in liver mortality and liver disease management, with a globally aligned curriculum flexible to local health systems. By developing and unifying a minimum standardised global curriculum we can achieve this goal, supporting minimum competencies for internationally accredited hepatologists and strengthening global knowledge transfer.

Precedents exist of a joint curriculum in gastroenterology (GI) and hepatology, including the European ‘Blue book’ which is a shared curricula for GI and hepatology standardisation, objective written examination for international trainees.^8^ Similarly, in medical oncology a global curriculum in 2004 was developed, with further revisions completed thus far.^9^ The success and subsequent uptake of this curriculum demonstrate that standardisation and a joint curriculum concept are effective and feasible.^10^ Hepatology curricula are constantly undergoing evolution;^11^, ^12^ where hepatology has recently been recognised as a sub-specialty in its own right, curricula and training are now being developed,^13^, ^14^, ^15^, ^16^ representing an ideal opportunity to harmonise educational standards. Furthermore, strategies to align clinical ability and objectively standardise knowledge could help enable trainees to gain experience in specialist placements that are not locally available, such as liver transplantation or liver intensive care. Such international training exchanges within a proposed curriculum model allows exposure to key conditions and treatment pathways, eg community diagnosis, tropical liver diseases, viral hepatitis therapy and genetic liver diseases that would not be otherwise experienced.

This study aims to compare existing international curricula and evaluate the feasibility of developing a shared global framework, ultimately leading to a global accreditation in hepatology. The primary objective is to identify core areas which potentially could be unified to help enhance the transferability and portability of training and ultimately enhance the quality of liver disease care across diverse health care settings.

## Methods

This is a cross-sectional study which compares the current hepatology curricula as of January 2024, endoscopic training requirements, procedural competencies, research outputs and assessments (both formative and summative) of regions across the world using the Cambridge assessment research division curriculum mapping framework.^17^ For an adequate initial feasibility comparison, we selected regions with courses which were taught and had curricula in English to prevent any language bias and ensured that there was representation from both LIC and HIC in order to allow adequate inclusion of populations and diseases. Requirements for selection included an accessible curriculum in English, representation across different continents, an adequate population and a rapidly changing healthcare system. The selected regions for the curriculum assessment were the United Kingdom (UK), United States of America (USA), Uganda, India, Bangladesh, Hong Kong, Canada and Australia (Hong Kong is a special administrative region of China, but does have its own training pathway and was therefore included in this assessment). This selection will represent approximately 25% of the world’s population and allowed representation across five continents.

The UK curriculum aligns with the European Blue Book for Gastroenterology and Hepatology training, enabling direct comparison with many European countries that follow the same standard. We elected to focus specifically on hepatology rather than gastroenterology due to the similar approach of detection, diagnosis, management alongside global health consequences of liver disease, features less consistently aligned across broader gastroenterology conditions.

Curricula and training pathways were provided by our consortia AA and JLY (UK), SS and TS (USA), PO (Uganda), AD (India), NK (Bangladesh), LM (Hong Kong), AF (Canada), JO’B (Australia), and official national training GI and hepatology societal documents available online. Review, comparison and collation of curricula were conducted by two independent assessors, with any differences in opinion regarding content decided by a third assessor (project lead). Data were collected in a binary form (eg present or not present) for curriculum items and other sections as applicable to create practical and comparable data. The remaining data were collected as numerical or categorical forms and are displayed in Table 1.

**Table 1:** Qualifications and assessments in current global hepatology training.

## Results

The curricula were divided into six sections, namely: curriculum core items, entry requirements, training pathways, research, procedural competency and competency assessment.

The goal of all these training pathways is unanimous, to produce independent or unsupervised clinicians able to provide competent gastroenterology and hepatology care.

### Curricula core items (Table 2)

The curricula show expected homogeneity, but Hong Kong lacks defined conditions as its viva exam aims to cover the entire GI disease spectrum. Without defined targets, we could not compare it with other regions.

All reviewed geographies shared non-clinical-based curricular targets: communication skills, team working, professional and ethical behaviour, teaching, ongoing education and healthcare resource management.

Many regions share similar clinical topics, such as acute liver failure, with some being better defined than others. The common curriculum points are presented in Table 2. Nomenclature for conditions has been stated as documented in curricula and combined appropriately where there have been changes over a time period (ie MASLD being the most notable).

**Table 2:** Current global hepatology core curriculum items.

Liver transplantation is mentioned in all curricula except Uganda and is an optional curriculum point for Hong Kong. There are variations from the umbrella term of liver transplant (Bangladesh) to specific points such as in the USA with transplant assessment, and pre-, post- and perioperative care including artificial liver support. All the curricula which have more than just an umbrella term specify experience in transplant assessment and post-transplant care.

### Entry requirements (Table 1)

There are three different entry routes into hepatology training for the regions reviewed. Bangladesh has direct entry from the end of medical school training requiring, therefore, only university qualification. Australia, Hong Kong, Uganda, Canada, India and UK have requirements for general medicine competencies before entry into GI training. The USA requires GI training before entry into the hepatology pathway.

### Training pathway (Fig. 1)

Only India has a dedicated hepatology training pathway independent of GI, while all the others have integrated GI and hepatology. Only the USA has a true alternate training pathway for hepatology while the UK has a semi-alternate training pathway, which we have deemed integrated. Canada, Australia, Uganda, Hong Kong and Bangladesh all have integrated GI and hepatology training, which are not distinct.

**Fig. 1:**
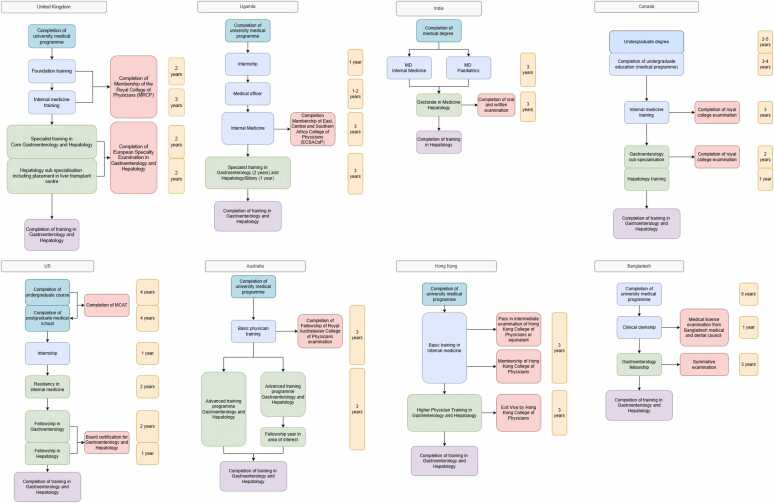
Current examples of global hepatology training pathways of UK, Uganda, India, US, Australia, Hong Kong, Canada and Bangladesh.

Applications for training are centralised in Australia, USA and India, and regional in the UK, while Canada, Uganda, and Bangladesh require individual applications to specific hospitals that have training schemes.

Length of hepatology training is not always specified. The Indian training programme is for 36 months, the UK specifies 24 months of hepatology, and the USA and Canadian programmes 12 months.

Additionally, USA has adopted a Fast-Track Pathway in Hepatology, an alternative training pathway designed for doctors who wish to pursue a career in transplant hepatology while shortening the total duration of training. This programme allows eligible candidates to complete their training in a total of 3 years, instead of the traditional 4 years.

### Research

All regions apart from Uganda have mention of research or scholarly requirements as part of the curriculum. The scope for what this entails varies from involvement in ‘a research project’ to publication in a peer-reviewed journal. In the Hong Kong curriculum it is specifically mentioned, but as an optional requirement and not mandatory for final accreditation. There is no formal requirement for a higher degree, eg PhD or MSc, in any of the programmes.

### Procedural competency

Endoscopic competency for oesophagogastroduodenoscopy (OGD) is the only requirement common to all regions that is necessary for hepatology training. Australia, Uganda and Bangladesh simply require independence in both diagnostic and therapeutic OGD. The remaining nations specify either experience treating oesophageal or gastric varices or that a trainee must perform a certain number of bandings, injection sclerotherapy or managing acute bleeding.

Abdominal paracentesis is an additional common procedure in which competency is also required. Only Bangladesh does not have this as a requirement for hepatology training. Liver biopsy and liver ultrasound is a required competency in India, Hong Kong and Bangladesh. India also includes transjugular liver biopsy and Bangladesh drainage of liver abscess as required competencies.

### Competency assessments (Table 1)

Trainees are assessed through a combination of clinical- based discussions / clinical examinations (CBD/CEX), direct observation of practice (DOP), logbooks, supervisor evaluations, and oral and written examination. Across all regions, at least two of these methods are used to assess trainees’ competency. Most regions use a combination of logbook and supervisor evaluations to assess competency for core curriculum items. In India and Hong Kong trainees must undergo an oral examination, which carries significant weighing in their overall assessment. UK and Australia require CBD/CEX and UK, USA and Uganda require DOP for assessment of competency. Canada has a written and oral examination required to achieve for competency, which they can only apply for with an institution’s approval.

On all reviewed curricula, the threshold for competency is not defined in regard to work-based assessments or evaluation. While it is necessary for a supervisor or faculty committee to conduct these assessments, the overall opinion on competency is not clearly defined.

Except for Australia, all regions require a national board exam to complete training. Only India and the USA have a standalone hepatology examination; others combine it with gastroenterology. The UK and Australia, which lack formal exit exams, use summative portfolio assessments instead.

## Discussion

Overall, while there is definite heterogeneity within hepatology curricula, there is sufficient homogeneity to support the concept of a global curriculum, reflecting the universal nature of liver disease with significant curriculum variations arising in response healthcare need, regional disease patterns and system capacity, underscoring the importance of a flexible, context-specific global training framework. For example, hepatologists in some areas will need to develop procedural/radiology competencies such as abdominal ultrasound and/or liver biopsy due to the otherwise relative lack of expertise and healthcare infrastructure. It would simply not be possible to keep the whole hepatology community at the same competency levels due to healthcare funding differences, most especially in less-resourced nations. The combined requirement for procedural ability is also reflected in the need for specific curriculum points. The Ugandan curriculum, for example, has limited gastroenterologists compared with the high disease presence. Therefore, the need to have an extensive and specific curriculum will ensure that trainees are meeting the required standards to cope with this broad burden of disease. Similarly, in Bangladesh, there is a specific training point on parasitic liver disease, reflecting local epidemiology and needs. While these conditions may be encountered in other regions such as UK or USA, their prevalence and significance are comparatively lower and less featured in training.

We reviewed the basic framework around competency assessment allowing most direct comparisons between curricula. Less objective assessments, however, make comparison difficult if similar tools are used to assess trainees, the goal remaining the same which should not prevent the use of subjective assessment. In terms of overall assessments, there is a significant gap, with the UK having a system based on supervisor evaluation undertaken gradually iteratively training, while Hong Kong has a system almost entirely based on an examination at the end of training. There is merit in both, with the Hong Kong examination conducted by senior doctors and focus on key areas of practice most relevant and best situated to be able to test trainee knowledge ensuring the required level needed to practise. The UK system is gradual, spread over a longer period, supporting trainees to develop their knowledge and skills, although it risks certain conditions being either overlooked or insufficiently assessed; while formal examination is included, it is not an ‘exit’ assessment, completed during training rather than end, limiting its role in ensuring uniform competency during completion.

**Fig. 2:**
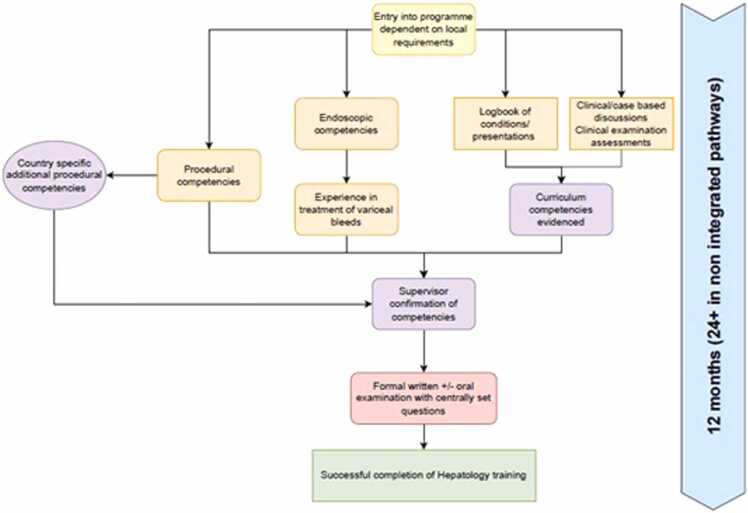
Example pathway for proposed global hepatology training.

This comparative work suggests adequate homogeneity across curricula and assessments to serve as a framework to form a global curriculum. Using the data that we have collected and reviewed, Fig. 2, Table 3, Table 4 are potential initial training frameworks, core list of curriculum conditions, areas that will need to be addressed to ensure homogeneity and practicality and rationale behind the design.

**Table 3:** Specific curriculum items in global hepatology training.

**Table 4:** Suggested recommendation and rationale for global hepatology training.

	Recommendation	Rationale
**Entry requirements**	Medical degree as a minimum requirement but experience in general medicine would be ideal	Due to the different needs in different countries, it would not be appropriate to make definitive general medical requirements for hepatology training. However, due to the overlap with internal medicine, certainly experience would be desirable and formal accreditation would not be detrimental.
**Length of training**	12 months minimum in an integrated pathway and 24 months minimum in non-integrated pathway. Wherever possible a minimum of 3 months of training should be spent in a centre where exposure to liver transplantation is possible.	The length of training needs to reflect a period in which it would be feasible to gain adequate exposure to hepatology, complete the necessary curriculum points and procedural competencies. In an integrated pathway this would be shorter since there will be exposure in non-dedicated training to help build experience.
**Exit examination**	Completion and achieving a pass on both written and oral examination set by an international exam board	This is a key component of the global curriculum, with the ability to objectively show that trainees have achieved the same standard of knowledge and training. A written examination will allow a reproducible and standardised way of ensuring this happens.
**Research**	Optional component, desirable but not required	There will be very significant variations in both access to research and having the necessary skills to conduct it. It would therefore be highly recommended for trainees to show participation in a hepatology research project, quality improvement work or audit during their training with appropriate review and feedback.
**Procedural competencies**	Required: Endoscopic competency in OGD, abdominal paracentesis	Different countries will have external requirements for competency in OGD which we would not seek to alter, we would suggest trainees should achieve national competency standards in OGD. Specifically we would suggest a minimum of 10% of necessary procedures/additional procedures should be therapeutic procedures in upper gastrointestinal bleeds. Trainees will need to provide evidence of cases to show exposure to variceal banding and sclerotherapy injection with supervisor sign-off of competency in these modalities which should make up 50% of their therapeutic procedures.
	Optional: Abdominal ultrasound, liver biopsy, liver biopsy interpretation	Optional procedures are reflective of the differing work conducted by hepatologists worldwide, eg Bangladesh, India, Hong Kong and Uganda with liver biopsy, as well as USA in liver ultrasound. In these cases competencies should be set by national or local standards.
**Assessment of competency**	Assessment based on combination of logbook, clinical/case-based discussions, supervised learning events with overall supervisor confirmation of competency	We combine both quantitative and qualitative assessment within this area to provide a broad overall assessment. Specific assessment will combine proof of exposure to hepatological conditions as well as specific discussions and learning events. Overall confirmation will be from a suitable supervisor to assess that the trainee has had adequate exposure to and understanding of the diseases/conditions from curriculum.
**Curriculum items**	Experience/exposure to all curriculum points.	Our approach to this would be to use a combination of specified conditions and umbrella terms to ensure there is adequate breadth of exposure. We would also use a combination of clinical situations as well as specific diseases.

These have been designed to account for the different entries from basic training pathways and for a formal assessment system incorporating different aspects of supervisors and formal examination. It also has an additional route for optional requirements, which can be country-specific to allow for more local needs. Some curriculum conditions appear broad, eg liver transplant, due to prevalence and burden of some conditions, while allowing for different approaches to reaching the required knowledge base and competencies. Training priorities may need to vary according to disease burden situations, eg geographical disparities in HCC, toxin exposure (aflatoxin) and rising dominance of metabolic dysfunction-associated steatotic liver disease (MASLD) in HIC. The curriculum will provide the essential knowledge to adequately treat these conditions; however, the utilisation of these skills will vary regionally to account for prevalence. The role of population level prevention and primary care becomes paramount in developing global health curriculum, incorporating competencies and experience in conditions with integrated pathways, primary care and cross-sector collaboration, including multidisciplinary chronic disease frameworks rather than single tertiary centres that could then be locally adapted. There is the ability within our model to streamline training together across different areas and the necessary flexibility to account for regional demographic variations, disease epidemiology and health system structure.

By providing regular updates to the curriculum, it will support trainees keeping up to date, including technological advancements, changes in practice, enhance outcomes and implement national diagnostic and management strategies. By introducing new concepts at a basic educational level, we would hope to allow the maximal uptake in clinical practice and standardisation with optimisation of services without necessary first-hand experience, for example LIC benefiting from lived experiences of specialist management pathways and HIC gaining experience in engagement and distribution of treatments across wide populations, helping to streamline services and reduce financial burden. As further work in this field is performed, it will also be necessary to involve national and international hepatology societies for their invaluable guidance and input on this topic.

There are clear limitations of our study as curriculum are not designed with the intention of comparison and within our methodology, whilst we strived to use a robust technique, there will be nuances that will not be captured accurately and translate to very different practices. Our curriculum data were based and taught in English, as a further potential bias, allowing the past and present influence and affiliations with the UK and current Commonwealth, yet demonstrating the presence of pre-existing potential framework that can be built on. The UK-based examinations, ie MRCP(UK), is an example of a globally accepted accreditation for internal medicine knowledge and is used by many regions as a benchmark of competency. Despite the obvious bias, our consortia of authors felt that as an initial proposed study, this selection of regions across continents covered different environmental and cultural backgrounds and with good representation globally, particularly since the UK remains aligned with the European Blue Book, and therefore comparable to participating European countries. We recognise significant gaps within the areas assessed, in particular South America and Asia. However, in this study we felt that the regions included were appropriate in the context of adult hepatology. We focused solely on adult hepatology while acknowledging a paediatric route into training. If our model is successful, it could potentially be expanded to include paediatrics.

Our work explores the potential of an adult global curriculum and should not be seen as an exhaustive list of competencies and knowledge needed to be a hepatologist merely as a minimum requirement to ensure a standard of base care to be provided. Countries and regions will have differing needs for their population and personalised adaptations including further specific training and assessments to ensure trainees have the necessary skills as independent practitioners.

In regions where hepatology exists within gastroenterology, often with poor delineation between those with and without additional hepatology accreditation, we suggest that the curriculum is considered as a continuous entity rather than binary (eg ‘basic’ or ‘advanced’). While not all clinicians will need to have extensive experience in liver transplant, an understanding of the role within the specialty will be necessary.

Given the global burden of liver disease, a unified curriculum will strengthen hepatology as a discipline, improving patient care by fostering a globally trained, highly competent hepatology workforce, thus facilitating international mobility for trainees. While our manuscript is not the final iteration, it supports developing a blueprint to improve global delivery of exemplary hepatology care by optimising trainee training and experience. Healthcare policy makers and regulatory authorities should therefore consider greater alignment and harmonising training standards, competencies as well and specialities definitions across geographies. Our intention is not to impose an absolute single global model but to encourage comparability and coherence allowing flexibility for context specific needs for local adaption.

## CRediT authorship contribution statement

**James C. Liu Yin:** Writing – review & editing, Writing – original draft, Visualization, Validation, Software, Resources, Project administration, Methodology, Investigation, Formal analysis, Data curation. **Matthew Parsons:** Writing – review & editing, Methodology, Data curation. **Ponsiano Ocama:** Funding acquisition, Conceptualization. **Osman Dar:** Writing – review & editing, Methodology. **Morro M.L. Touray:** Writing – review & editing, Methodology. **James O'Beirne:** Writing – review & editing. **Thomas Schiano:** Writing – review & editing, Methodology, Conceptualization. **Aftab Ala:** Writing – review & editing, Validation, Supervision, Project administration, Methodology, Formal analysis, Conceptualization. **Ajay Duseja:** Writing – review & editing, Methodology. **Sanjaya Satapathy:** Writing – review & editing, Validation, Resources.

## Ethics approval and consent to participate

Ethics approval was not required for the study as no human participants were involved and only secondary data that is publicly available have been used.

## Funding

The authors declare that no funds, grants, or other support were received during the preparation of this manuscript. MML Touray is a member of NIHR Applied Research Collaboration – Kent, Surrey and Sussex (ARC-KSS) and utilised allocated research time from this programme to contribute to this work. AA Aftab Ala is supported by the National Institute of Health and Care Research.

## Declaration of competing interest

The authors declare that they have no known competing financial interests or personal relationships that could have appeared to influence the work reported in this paper.

## Data Availability

The data used will be shared with partners upon reasonable request to the corresponding author.
